# Risk Factor Analysis of Mechanical Complications in Surgical Treatment of Thoracolumbar Deformity with Osteoporotic Vertebral Fracture

**DOI:** 10.3390/jcm13247618

**Published:** 2024-12-13

**Authors:** Yoshihisa Kotani, Takahiro Tanaka, Atsushi Ikeura, Takanori Saito

**Affiliations:** 1 Spine and Nerve Center, Department of Orthopaedic Surgery, Kansai Medical University Medical Center, Moriguchi 570-8507, Osaka, Japan; takahiro.1124.t.t@gmail.com (T.T.); a_ikeike55@mac.com (A.I.); 2 Department of Orthopaedic Surgery, Kansai Medical University Hospital, Hirakata 573-1191, Osaka, Japan; saitot@hirakata.kmu.ac.jp

**Keywords:** risk factor, adult spinal deformity, osteoporotic vertebral fracture, spinal reconstruction, mechanical complication

## Abstract

**Objective:** Adult spinal deformity (ASD) with osteoporotic vertebral fractures (OVF) often requires vertebral body resection and replacement. However, postoperative mechanical complications (MC) have been unsolved issues. This study retrospectively investigated the risk of MC following anterior-posterior spinal fusion (APF) with vertebral body resection and replacement for OVF with ASD. **Methods:** Among 91 cases undergoing APF with vertebral body resection and replacement, 43 cases met the deformity criteria. The mean age was 74.2 years, and the mean number of fused segments was 5.7. Pre and postoperative spinal alignments were measured, and the risk of MC occurrence, including PJK, DJK, and cage sinking, was determined through multivariate analysis. The AUC and cutoff values were calculated through ROC analysis. **Results:** The incidence of MC, PJK, and DJK were 28%, 12%, and 14%, respectively. Multivariate analysis for MC revealed postoperative PI-LL and operative time (cutoff: 40.5 degrees, 238 min) as significant risk factors, while postoperative PI-LL was a significant risk factor for PJK (cutoff: 42.4 degrees). Evaluation considering only thoracolumbar level showed postoperative local kyphosis as a significant MC risk factor (cutoff: 11 degrees). There was a positive correlation between operative time and preoperative local kyphosis, with a cutoff value of 238 min being equivalent to 21 degrees. **Conclusion:** The postoperative mismatch over 40 degrees and preoperative local kyphosis over 21 degrees were considered as a high risk for MCs. The postoperative kyphosis of 11 degrees was the risk factor of MC in the thoracolumbar level. The meticulous preoperative assessment, including local and global alignment, and local flexibility as well as detailed surgical planning of fixation range and the requirement of osteotomy, are crucial.

## 1. Introduction

Due to the increase of aging populations, osteoporotic vertebral fracture (OVF) has become the frequent problem in many countries and has brought a significant impact on patients’ function, quality of life, and medical costs [1,2,3,4,5]. The primary treatment option is conservative. However, OVF with intractable pain, neurologic symptoms, pseudarthrosis, or severe progressive deformity requires surgical treatment. There are many surgical modality options, such as anterior, posterior, and combined anterior and posterior surgery [1,2,3,4,5]. Hosogane et al. surveyed the series of OVF surgeries comprising 403 patients [1]. It consisted of 87% posterior surgery, 8.7% anterior-posterior combined, and 4.7% anterior surgeries. The perioperative complications and mechanical complications (MC) consisting of screw loosening, screw backout, and proximal and distal junctional kyphosis (PJK, DJK) were 18.1% and 41.2%, respectively. Watanabe et al. surveyed 315 OVF patients and compared five different surgical modalities of anterior, posterior with or without osteotomy, anterior-posterior combined, and posterior with vertebroplaty [5]. The perioperative complications were 7.4 to 26%, and mechanical complications ranged from 14.8–26.3%.

In turn, the risk factor for mechanical complication was investigated by several authors [4,6,7]. Kudo et al. investigated 31 patients of OVF with kyphosis over 40 degrees who received posterior surgery with osteotomy or anterior-posterior surgeries [4]. The proximal and distal junctional kyphosis (PJK, DJK) were demonstrated in 16% and 35.5%, respectively, and 54% of patients required the fusion extension to pelvis. They found the significant difference of sagittal vertebral axis (SVA), lumbar lordosis (LL), lower LL (LLL), pelvic tilt (PT), and pelvic incidence (PI)-LL between DJK and non-DJK patients. Tamai et al. investigated 403 patients of OVF with neurologic deficits who received corrective fusion surgeries [7]. The risk for proximal junctional fracture was demonstrated in patients with osteoporosis and fusion to sacrum. 

Recently, several minimally invasive surgical modalities were applied to OVF treatment. Instead of classical anterior corpectomy and reconstruction or posterior three-column osteotomies [8], minimally invasive corpectomy and vertebral body reconstruction followed by percutaneous pedicle screw (PPS) fixation has become a choice of surgical treatment for severe OVF with local kyphosis [2,9]. There was a paucity of information whether the recent MIS corpectomy and vertebral body reconstruction followed by PPS fixation effectively decreases the occurrence of mechanical complication (MC) in patients with combined OVF and kyphotic spinal deformity. The objectives of this study were to retrospectively investigate the occurrence and details of mechanical complication in MIS corpectomy and vertebral body reconstruction followed by PPS fixation for OVF with kyphotic deformity, and secondary, to assess the risk factor for MC statistically.

## 2. Materials and Methods

From April 2014 to March 2023, a total of 91 thoracolumbar OVF were treated by minimally invasive anterior-posterior combined surgery with vertebral body replacement [5,8,9]. Among them, the following criteria defining spinal deformity was applied (Pelvic tilt (PT) > 22-degree, SVA > 50 mm, and PI-LL > 12 degrees) and 43 patients were consequently selected for this study [10]. There were 10 males and 33 females with average age of 74.2 years old (47–87). The average body mass index was 22.7 kg/m^2^ (13.7–50.5). The level of OVF was T12 or L1 in 27 patients, L2 or L3 in 12 patients, and L4 in four patients. The surgery included anterior corpectomy and artificial vertebral body replacement via minimally invasive approach followed by percutaneous pedicle screw fixation (PPS) either in lateral decubitus or prone position. 

### 2.1. Description of Surgical Procedure

The patient was placed in the right decubitus position. The four centimeters transverse incision was made two fingers anterior to the mid-axillary line, and the retroperitoneal space was enlarged to expose the psoas muscle. In the thoracolumbar junction, the diaphragm was partially dissected. The psoas muscle was retracted posteriorly to expose the lateral part of the fractured vertebral body and adjacent discs. After the retractor was placed, the fractured vertebral body was resected with adjacent intervertebral discs followed by careful removal of cartilaginous end plates. The artificial expandable vertebral body (T2 Altitude, Medtronic, or Xcore2, Nuvasive, San Diego, CA, USA) was placed under manual kyphosis correction force in conjunction with autograft and demineralized bone matrix (DBM). T2 Altitude (20 × 35–40 mm footprint) was used in 13 patients, and Xcore2 (18 × 35–40 mm footprint) was used in 30 patients. In thoracolumbar OVF, the caudal one-level OLIF was added followed by two-above and two-below PPS fixation. In lumbar OVF below L3, two-level posterior fixation was performed regularly. When large SVA or PI-LL mismatch existed preoperatively, the long-segment fixation was selected with or without pelvic fixation to correct the global balance. In the posterior fixation procedures, no augmentation procedures such as cement screws, hooks, or sublaminar wiring were used. As most patients were introduced from other municipal or private hospitals for surgery, no preceding outpatient treatment including osteoporosis treatment was performed. After surgery, most patients received osteoporosis treatments such as biweekly parathyroid hormone or romosozumab injections. 

### 2.2. Radiologic Evaluations

The following parameters were measured in standing radiographs preoperatively and at 1 year postoperatively: local kyphosis (LK) measured between the upper endplate of the cephalad vertebra and the lower end plate of the caudal vertebra to the fractured vertebra; thoracic kyphosis between T5 and T12 (TK); coronal vertical axis (CVA); SVA; PI; PT; LL; and PI-LL. The spinal fusion was assessed with CT at 1 year postoperatively. The bone bridging between the cephalad and the caudal vertebra shown in both coronal and sagittal sections without screw loosening was regarded as solid fusion at 1 year postoperatively. The PJK was defined as the sagittal Cobb angle over 10 degrees between the upper instrumented vertebra and the two-cephalad adjacent vertebra [11]. The DJK was defined as the sagittal Cobb angle over 10 degrees between the lower instrumented vertebra and the two-caudal adjacent vertebra. The mechanical complications including PJK, DJK, screw or rod breakage, adjacent vertebra fracture, and screw loosening were recorded. 

### 2.3. Statistical Analysis Procedures

To elucidate the risk factors for mechanical complication (MC), multivariate analysis was conducted with the use of EGR software (R version 4.2.2, The R foundation for statistical computing, Vienna, Austria, 2022). The logistic regression analysis with the variable reduction method was conducted with the objective variable of occurrence of MC, PJK, and DJK, respectively. The explanatory variables included a fracture level, fixation range, operation time (OT), preoperative and follow-up LK, preoperative and follow-up TK, preoperative and follow-up PT, preoperative and follow-up SVA, preoperative and follow-up CVA, preoperative and follow-up LL, and preoperative and follow-up PI-LL. The analysis was conducted in whole OVFs followed by the analysis only at the thoracolumbar levels (T12 and L1). After the multivariate analysis, the receiver operating characteristic (ROC) analysis was performed between the objective variables and the statistically significant explanatory variables. The area under curve (AUC) and cutoff value were obtained for each analysis. This study was conducted based on the acceptance of the ethical university committee, and patient data and radiographs without personal information in this report were shown the consent by patients.

## 3. Results

**Figure 1:**
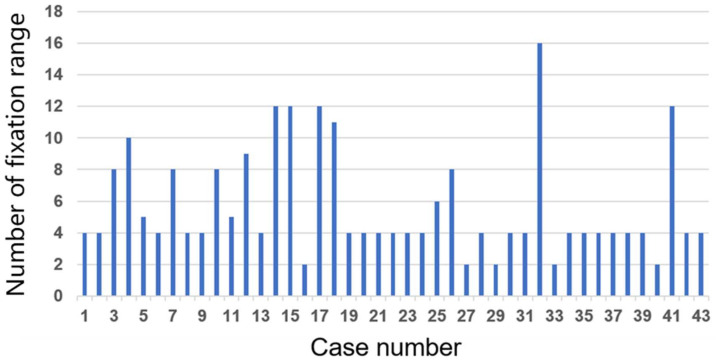
Fixation range with respect to each case. Horizontal axis: case number; Vertical axis: number of fixation range.

The average fixed segments were 5.7 segments (2–16), and the fixation range in each case was demonstrated in Figure 1. The average oblique lateral interbody fusion (OLIF) segments were 2.6 segments (1–6). The average operation time was 333.9 min (154–798), and the average estimated blood loss was 498.6 mL (5–2644). The solid fusion was confirmed in 41 out of 43 patients (95%). The mechanical complications (MC) were detected in 12 patients out of 43 patients (27.9%). The PJK and DJK occurred in five (11.6%) and six (13.9%) patients, respectively. One case demonstrated a set screw detachment. The five cases of revision surgeries were required: fusion extension in four; and balloon kyphoplsty in one. The average local kyphosis (LK) was 24.1 degrees preoperatively (−6–59) and corrected to 3.3 degrees (−22.7–50) postoperatively, which was statistically different at the *p* < 0.01 level (Table 1). The average TK was 33.6 degrees preoperatively (−21.7–94.0) and corrected to 33.6 degrees postoperatively (−1.4 to 88.0), which was statistically equivalent (NS). The average CVA was 22.9 mm preoperatively (0–100.3) and corrected to 17.1 mm postoperatively (0–56), which was statistically equivalent (NS). The average SVA was 117.3 mm preoperatively (−9–308.1) and corrected to 89.1 mm postoperatively (−24.3–756), which was statistically equivalent (NS). The average PI was 52.9 degrees (31.5–79.7) preoperatively. The average PT was 29.5 degrees preoperatively (4.5–50.5) and corrected to 25.3 degrees postoperatively (4.0–54.5), which was statistically different at the *p* < 0.03 level. The average LL was 21.8 degrees preoperatively (−22.9–74.4) and corrected to 33.8 degrees postoperatively (1.0–53.0), which was significantly different at the *p* < 0.01 level. The average PI-LL mismatch was 33.2 degrees preoperatively (−22.4–91.6) and corrected to 20.2 degrees postoperatively (−16.0–52.8), which was significantly different at the *p* < 0.01 level. 

Multivariate analysis demonstrated that postoperative PI-LL mismatch and operation time (OT) were significant risk factors for mechanical complications (PI-LL: *p* = 0.049; OR:1.08; OT: *p* = 0.01; OR:1.01) (Table 2). The separate analysis for PJK demonstrated that postoperative PI-LL mismatch was a significant risk factor at the *p* = 0.039 level (OR: 1.01). The separate analysis for DJK did not show any significant risk factors. When the OVF levels were confined to thoracolumbar level (T12 and L1), postoperative local kyphosis was the significant risk factor for mechanical complications (*p* = 0.041, OR: 1.22).

**Figure 2:**
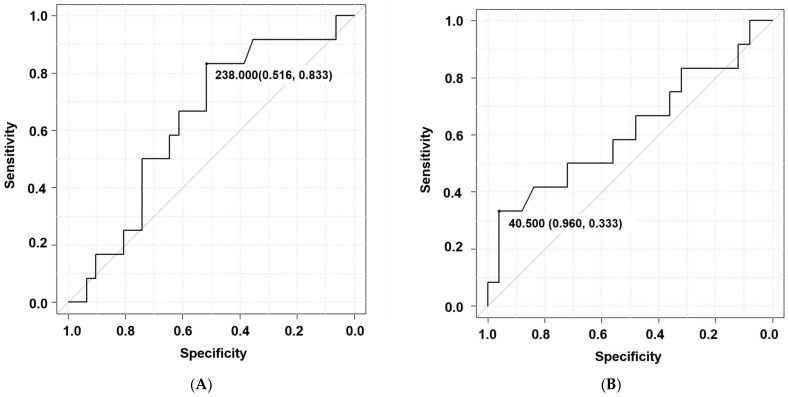

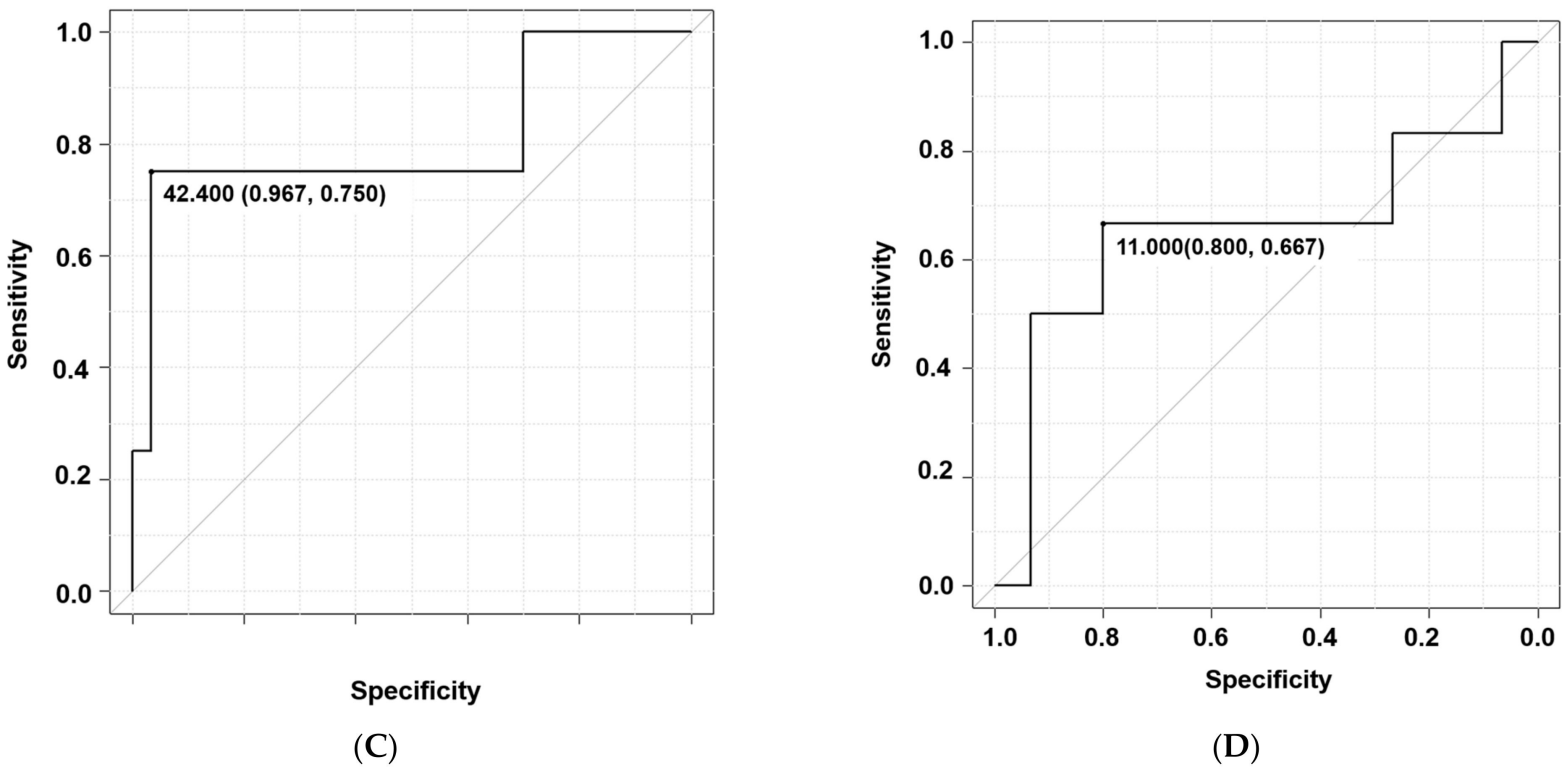
Receiver Operating Characteristic (ROC) curve based on multivariate analysis. (**A**): The analysis between mechanical complication and operation time demonstrated the area under the curve (AUC) of 0.633 (0.452–0.814) with the cutoff value of 238 min. (**B**): The analysis between mechanical complication and postoperative PI-LL demonstrated the area under the curve (AUC) of 0.615 (0.401–0.829) with the cutoff value of 40.5 degrees. (**C**): The analysis between PJK and postoperative PI-LL demonstrated a high AUC value of 0.808 (0.471–1) with the cutoff value of 42.4 degrees. (**D**): The confined analysis in thoracolumbar OVF between mechanical complication and postoperative local kyphosis demonstrated the area under the curve (AUC) of 0.656 (0.326–0.985) with the cutoff value of 11.0 degrees.

The additional ROC analysis was performed based on the multivariate analysis. The analysis between mechanical complication and operation time demonstrated the AUC of 0.633 (0.452–0.814) with the cutoff value of 238 min (Figure 2A). The analysis between mechanical complication and postoperative PI-LL demonstrated the AUC of 0.615 (0.401–0.829) with the cutoff value of 40.5 degrees (Figure 2B). The analysis between PJK and postoperative PI-LL demonstrated a high AUC value of 0.808 (0.471–1) with the cutoff value of 42.4 degrees (Figure 2C). The confined analysis in thoracolumbar OVF between MC and postoperative LK demonstrated the AUC of 0.656 (0.326–0.985) with the cutoff value of 11.0 degrees (Figure 2D).

**Figure 3:**
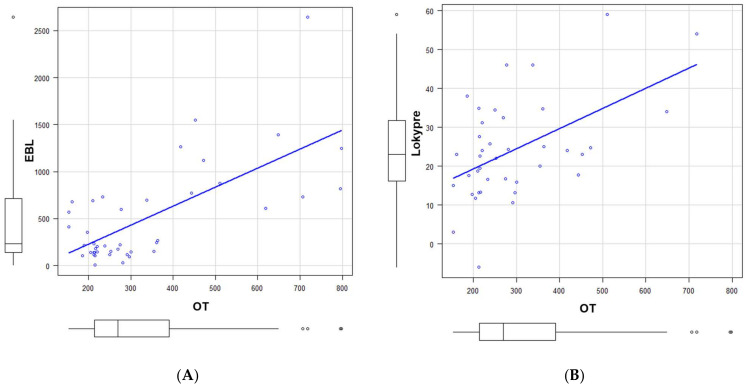
Linear regression analyses between operation time and other parameters. (**A**): Correlation between operation time (OT) and estimated blood loss (EBL) with correlation coefficient of 0.691. (**B**): Correlation between operation time (OT) and preoperative local kyphosis (Lokypre) with correlation coefficient of 0.514.

As the multivariate analysis showed the operation time for the risk factor of MC, we conducted additional linear regression analyses between operation time and other parameters (Table 3). The results demonstrated that the estimated blood loss, number of fixed spinal segments, and preoperative LK significantly correlated to operation time with correlation efficients of 0.691, 0.873, and 0.514, respectively (Table 3, Figure 3A,B). To obtain the preoperative LK value corresponding to the OT cutoff value of 238 min in the multivariate analysis, we conducted an additional simple regression analysis. The obtained formula was as follows: Preoperative LK = 0.0519 × OT + 8.891 (*p* = 0.0007). Consequently, a preoperative LK of 21 degrees was the corresponding value of the OT cutoff value of 238 min.

### 3.1. Case Presentations

#### 3.1.1. Case 1: 84 Years Old, Female, L1 Vertebral Collapse

**Figure 4:**
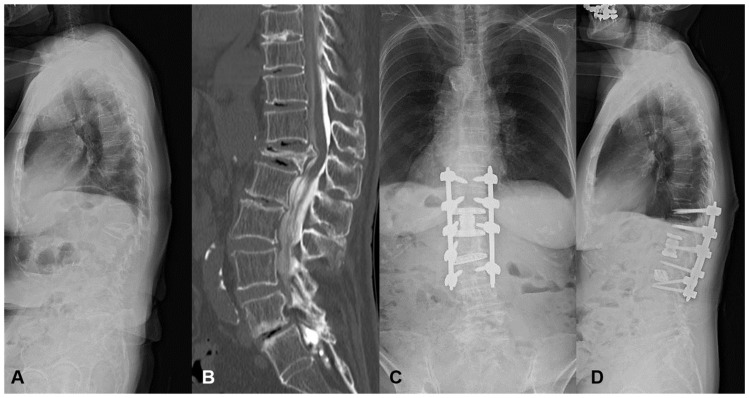
An 84-year-old female suffering from L1 vertebral collapse. The L1 MIS corpectomy, artificial body replacement, and L2/3 XLIF were performed followed by PPS fixation. The patient became ambulatory, and motor weakness recovered without mechanical complications. (**A**) Preoperative lateral X-ray, (**B**) Preoperative myelogram CT, (**C**) Standing AP X-ray at one year postoperatively, (**D**) Standing lateral X-ray at one year postoperatively.

She revealed severe motor weakness of right quadriceps, drop foot, and gait disturbance preoperatively. The L1 MIS corpectomy, artificial body replacement, and L2/3 XLIF were performed followed by PPS fixation. The local kyphosis of 24 degrees was corrected to 0 degrees postoperatively. Even with a high PI of 66 degrees, no mechanical complication occurred with the enhancement of the fixation level (Figure 4). The patient became ambulatory with the recovery of motor weakness. 

This case was a typical example of thoracolumbar OVF with preoperative high mismatch. When the short-segment fusion was conducted, there was a very high risk for MC such as DJK.

#### 3.1.2. Case 2: 77 Years Old, Male, L1 Vertebral Collapse

**Figure 5:**
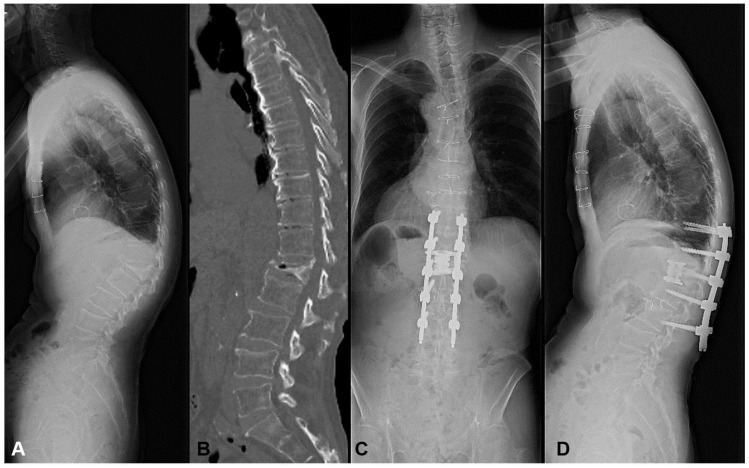
A 77-year-old male suffering from L1 vertebral collapse. The MIS L1 corpectomy, artificial body replacement, and L2/3 OLIF were performed followed by single-position percutaneous screw fixation. However, the distal screw pulled out at 1 month postoperatively. (**A**) Preoperative lateral X-ray, (**B**) Preoperative CT, (**C**) Standing AP X-ray at one month postoperatively, (**D**) Standing lateral X-ray at one month postoperatively.

He suffered from a progressive L1 vertebral collapse after a compression fracture by falling down. Due to intractable pain, he was unable to walk without a support preoperatively. The MIS L1 corpectomy, artificial body replacement, and L2/3 OLIF were performed followed by single-position percutaneous screw fixation (Figure 5). Even the LK of 42 degrees and PI-LL of 16 degrees were corrected to 19 degrees and 8 degrees, respectively. However, the distal screw pulled out at 1 month postoperatively. The patient required hardware removal after the complete fusion at 1 year postoperatively. 

In this case, we conducted additional enhancement of L2/3 OLIF to prevent MC. However, the local kyphosis was not corrected efficiently (19 degrees). This exceeded our obtained threshold of 11 degrees, leading to DJK in this case.

#### 3.1.3. Case 3: 78 Years Old, Female, L4 Vertebral Collapse

**Figure 6:**
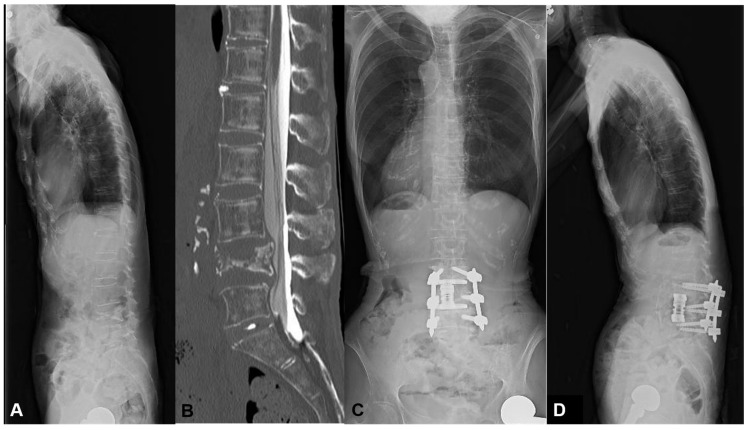
A 78-year-old female suffering from L4 vertebral collapse. The MIS L4 corpectomy followed by percutaneous screw fixation was conducted, resulting in the recovery of motor loss and gait disturbance without mechanical complications. (**A**) Preoperative lateral X-ray, (**B**) Preoperative myelogram CT, (**C**) Standing AP X-ray at one year postoperatively, (**D**) Standing lateral X-ray at one year postoperatively.

The patient suffered from L4 compression fracture 4 months ago. The gait disturbance progressed with the weakness of lower extremities due to neural compression of vertebral collapse. The MIS L4 corpectomy followed by percutaneous screw fixation was conducted, resulting in the recovery of motor loss and gait disturbance. (Figure 6) In this case, we conducted short-segment fusion because of no LK and low PI of 42 degrees. After surgery, the LK was zero degrees with a postoperative mismatch of 30 degrees. These values were within our obtained threshold of 40 degrees, leading to no MC. 

## 4. Discussion

The surgical treatment of adult spinal deformity involved several perioperative and postoperative complications [12,13,14]. Smith et al. reported a total of 70% complication rates in posterior-based ASD surgeries, with 52% at the perioperative and 43% at the delayed phases [12]. Sciubba et al. investigated 11,692 ASD patients and reported a 55% overall complication rate [13]. Among several perioperative and postoperative complications, the mechanical complications included screw pullout, fracture of adjacent vertebra, PJK, and DJK, often requiring revision surgery. In the treatment of OVF, Hosogane et al. reported that 403 OVF patients requiring the surgery resulted in 41.2% of mechanical complications and 6.2% of pseudarthrosis [1]. The increased occurrence of mechanical complication in OVF combined with thoracolumbar kyphosis was reported by Kudo et al. [4]. In this study, 16% of PJK and 35.5% of DJK were reported when thoracolumbar kyphosis exceeded 40 degrees. When comparing the DJK group and non-DJK group, significant differences were demonstrated in SVA, LL, PT, and PI-LL. The greater sagittal imbalance and larger mismatch, and PT patients tended to demonstrate mechanical complications. 

Several studies investigated the risk factors for reconstruction surgeries of either OVF or ASD [4,5,6]. However, no study investigated the risk factor of MIS reconstruction surgeries for combined OVF and ASD. Also, there was a paucity of information regarding the difference in risk factor between thoracolumbar and lumbar level. We analyzed the risk factor at the thoracolumbar and lumbar level separately and consequently obtained unique results over previous reports [4,5,6].

In the present study, the risk factor of mechanical complication in the surgical treatment of spinal deformity combined with OVF was investigated. The definition of spinal deformity in this study was set at PT > 22 degrees, SVA > 50 mm, and PI-LL > 12 degrees, described by Schwab et al. [10]. This threshold was explained at the level in which the quality of life of patients was significantly impaired using patient-reported quality of life parameters. Under this study setup, the occurrence of MC, PJK, and DJK was 27.9%, 11.6%, and 13.9%, respectively, which was much smaller than those in previous reports [1,3,4]. This may be explained by the surgical procedure in this study in which minimally invasive corpectomy and posterior percutaneous pedicle screws were utilized. The minimally invasive anterior and posterior procedures minimized a rib resection, diaphragm resection, and ligamentous and muscular damages, contributing to the preservation of biomechanical stability of the spinal structure. Even using a MIS surgical modality, the initial correction of kyphotic deformity was sufficient, in which the preoperative LK of 24.1 degrees was corrected to 3.3 degrees on average, postoperatively.

The risk factors of MC detected in this study were operation time of 238 min, postoperative mismatch of 40.5 degrees, and postoperative thoracolumbar kyphosis of 11 degrees or more. The risk factor of PJK was postoperative mismatch of 42.5 degrees or more. To date, many studies investigated the risk factors for MC in the surgical treatment of OVF. Kudo et al. conducted ROC analysis between DJK and non-DJK groups, demonstrating cutoff values of 80 mm in SVA, 18 degrees in LL, 23 degrees in mismatch, and 28 degrees in PT [4]. They mostly highlighted the global sagittal and spino-pelvic parameters. However, no local deformity parameters were demonstrated. Tamai, et al. investigated 403 patients of OVF with neurologic deficits, and reported 15.6% of proximal junctional fracture [7]. They demonstrated that osteoporosis and fusion to sacrum were risk factors of MC. The cutoff value of bone mineral density was 0.61 g/cm^2^. Du et al. investigated 88 patients using a predictive scoring system in the posterior surgery for OVF [6]. PJK was detected in 25 patients (28.4%). The risk factor of PJK was demonstrated in patients 70 years old or older with a BMI greater than 28 kg/m^2^, a PI-LL larger than 20 degrees, and the existence of posterior ligamentous complex injury. Our study detected local kyphosis and postoperative PI-LL mismatch as the parameter of risk factors. However, there were no statistical relationships with age or global sagittal parameters. 

In the present study, we employed several parameters in addition to radiologic parameters for multivariate analysis of risk factor analysis. Specifically, age, sex, BMI, OVF level, fixation range, number of OLIF, and fusion status were included. However, only radiologic parameters and operation times were statistically significant risk factors. The major limitation of this study was an inability to include osteoporosis parameters for multivariate analysis. This was because most candidates of this study were introduced from other local or private hospital only for the purpose of operation. Therefore, any preceding outpatient evaluations were lacking. Further study will evaluate DEXA or the vertebral Hounsfield unit (HU) using preoperative CTs for the osteoporosis parameter. Another limitation of this study lay in the lack of flexibility evaluation in the kyphotic deformity. The rigidity of kyphotic deformity varies with patients, and the inclusion of flexibility parameters was ideal in the risk factor analysis. However, most OVF patients with deformity were in a lot of pain and were unable to receive functional radiologic evaluation preoperatively. Other study limitations included a retrospective study design, causing a selection bias, a limited number of participants without power analysis, and a regional bias due to the limited location of the hospital.

From the results of the multivariate analysis, we reconsidered the meaning of operation time as the risk factor. We conducted additional linear regression analysis (Table 2). According to the results, estimated blood loss, the number of fixed spinal segments, and preoperative LK were significant factors related to operation time. We assumed that large LK cases are mostly stiffer than smaller cases and that the anterior release procedure including corpectomy simply requires more time and bleeding in those cases. Therefore, these factors are closely related to MC due to the magnitude and rigidity of LK. 

The results of this study suggested the importance of preoperative radiologic evaluation in the treatment of spinal deformity with OVF. When the LK at the OVF exceeded 21 degrees, it was necessary to consider the possibility of correcting the kyphosis only by corpectomy or by the additional posterior osteotomy required. The preoperative flexibility evaluation of fulcrum backward bending helps this assessment. The bridging bone formation at the anterior edge of OVF sometimes makes the deformity rigid, which requires anterior osteotomy to the contralateral side of approach or posterior Grade 2 osteotomy. The present study also emphasized the consideration of preoperative and postoperative PI-LL mismatch to prevent mechanical complication as well as PJK occurrence. When the postoperative mismatch is estimated to become larger than 40 to 42 degrees, it has a high possibility of causing MC or PJK. As the fixation range was not related to the occurrence of MC, other additional modalities of adjacent lateral interbody fusion with high lordotic cage, or multiple grade 2 osteotomies, should be preferable. 

## Figures and Tables

**Table 1:** Summarized radiologic results.

	Preop	Postop	Significance
Ave. local kyphosis (LK; deg)	24.1 (−6–59)	3.3 (−22.7–50)	*p* < 0.01
Ave. thoracic kyphosis (TL; deg)	33.6 (−21.7–94)	33.6 (1.4–88)	NS
Ave. CVA (mm)	22.9 (0–100.3)	17.1 (0–56.0)	NS
Ave. SVA (mm)	117.3 (−9–308.1)	89.1 (−24.3–756.0)	NS
Ave. PT (deg)	29.5 (4.5–50.5)	25.3 (4.0–54.5)	*p* < 0.03
Ave. LL (deg)	21.8 (−22.9–74.4)	33.8 (1.0–53.0)	*p* < 0.01
Ave. PI-LL (deg)	33.2 (−22.4–91.6)	20.2 (−16.0–52.8)	*p* < 0.01

Remarks: The average number and range were depicted in each category. Statistical difference was shown on right column. NS: no statistical difference, Preop: Preoperative, Postop: Postoperative, CVA: coronal vertebral axis, SVA: sagittal vertebral axis, PT: pelvic tilt, LL: lumbar lordosis, PI: pelvic tilt.

**Table 2:** Multivariate analysis results.

Object Variable	Explanatory Variable	Odd Ratio	95% Confidence Interval	*p* Value
MCP	Mismatchpost	1.08	1.00–1.17	0.0491
MCP	OT	1.01	1.01–1.02	0.0170
PJK	Mismatchpost	1.01	1.00–1.02	0.039
DJK	NA	NA	NA	NS
MCP(TL)	Lokypost	1.22	1.01–1.470	0.041

Remarks: MCP: Mechanical complication, PJK: Proximal junctional kyphosis, DJK: Distal junctional kyphosis, TL: Thoracolumbar level, Mismatchpost: Postoperative mismatch (PI-LL), OT: Operation time, Lokypost: Postoperative local kyphosis, NA: Not applicable, NS: no significance.

**Table 3:** Relationship between operation time (OT) and other parameters.

	Correlation Coefficient	95% Confidence Interval	*p* Value
Estimated blood loss	0.691	0.492–0.821	0.000000297
Preop local kyphosis	0.514	0.239–0.715	0.000778
Postop local kyphosis	0.184	−0.149–0.479	0.277
Preop TK	0.269	−0.0423–0.532	0.0892
Postop TK	0.199	−0.12–0.481	0.218
Preop CVA	0.284	−0.0262–0.544	0.0722
Postop CVA	−0.2	−0.475–0.111	0.205
Preop SVA	0.213	−0.102–0.488	0.182
Postop SVA	−0.107	−0.398–0.203	0.498
PI	−0.124	−0.409–0.184	0.43
Preop PT	0.136	−0.179–0.426	0.396
Postop PT	−0.282	−0.539–0.0243	0.0707
Preop LL	−0.219	−0.488–0.0866	0.157
Postop LL	0.244	−0.0643–0.51	0.119
Preop PI-LL	0.182	−0.125–0.457	0.243
Postop PI-LL	−0.251	−0.516–0.057	0.108

Remarks: TK: Thoracic kyphosis; CVA: Coronal vertical axis; SVA: Sagittal vertical axis; PI: Pelvic incidence; PT: Pelvic tilt; LL: Lumbar lordosis.

## Data Availability

The data presented in this study are available in this article.
